# Publisher Correction: GIANA allows computationally-efficient TCR clustering and multi-disease repertoire classification by isometric transformation

**DOI:** 10.1038/s41467-021-25693-2

**Published:** 2021-09-03

**Authors:** Hongyi Zhang, Xiaowei Zhan, Bo Li

**Affiliations:** 1 Lyda Hill Department of Bioinformatics, UT Southwestern Medical Center, Dallas, TX USA; 2 Department of Population and Data Science, UT Southwestern Medical Center, Dallas, TX USA; 3 Department of Immunology, UT Southwestern Medical Center, Dallas, TX USA

**Keywords:** Genome informatics, Adaptive immunity

Correction to: *Nature Communications* 10.1038/s41467-021-25006-7, published online 4 August 2021.

In the original version of this Article Equation 13 was mistakenly formulated as:

$${{{{{{\boldsymbol{\Omega }}}}}}}_{6}=\left(\begin{array}{lll}{\underline{\underline{0}}} & {\underline{\underline{0}}} & {{{{{{\boldsymbol{\Omega }}}}}}}_{2} \\ {{{{{{\boldsymbol{\Omega }}}}}}}_{2} & {\underline{\underline{0}}} & {\underline{\underline{0}}}\\ {\underline{\underline{0}}} & {{{{{{\boldsymbol{\Omega }}}}}}}_{2} & {{{{{{\boldsymbol{\Omega }}}}}}}_{2}\end{array}\right)$$

The correct formulation is:

$${{{{{{\boldsymbol{\Omega }}}}}}}_{6}=\left(\begin{array}{lll}{\underline{\underline{0}}} & {\underline{\underline{0}}} & {{{{{{\boldsymbol{\Omega }}}}}}}_{2}\\ {{{{{{\boldsymbol{\Omega }}}}}}}_{2} & {\underline{\underline{0}}} & {\underline{\underline{0}}}\\ {\underline{\underline{0}}} & {{{{{{\boldsymbol{\Omega }}}}}}}_{2} & {\underline{\underline{0}}}\end{array}\right)$$

This has been corrected in both the HTML and PDF versions of the Article.

