# Supplementary material for: GIANA allows computationally-efficient TCR clustering and multi-disease repertoire classification by isometric transformation
Source: Nat Commun. 2021 Aug 4;12:4699. doi: 10.1038/s41467-021-25006-7 (PMC8339063; doi:10.1038/s41467-021-25006-7)
Supplement: Supplementary file 1 — Supplementary Information [file 41467_2021_25006_MOESM1_ESM.pdf]

Supplementary Figures

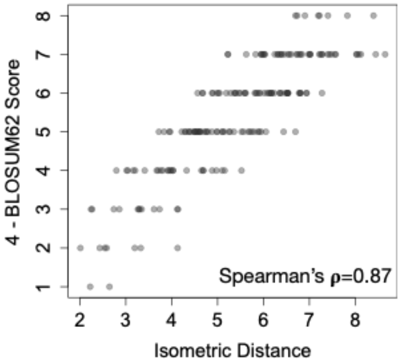

Figure S1. Performance of MDS-based isometric embedding. Euclidean distances (squared) between pairs of amino acids were calculated, and compared to the corresponding transformed BLOSUM62 dissimilarity scores (4-BLOSUM62 scores, with diagonal set 0). Spearman's correlation was calculated to evaluate the similarity of the two measures.

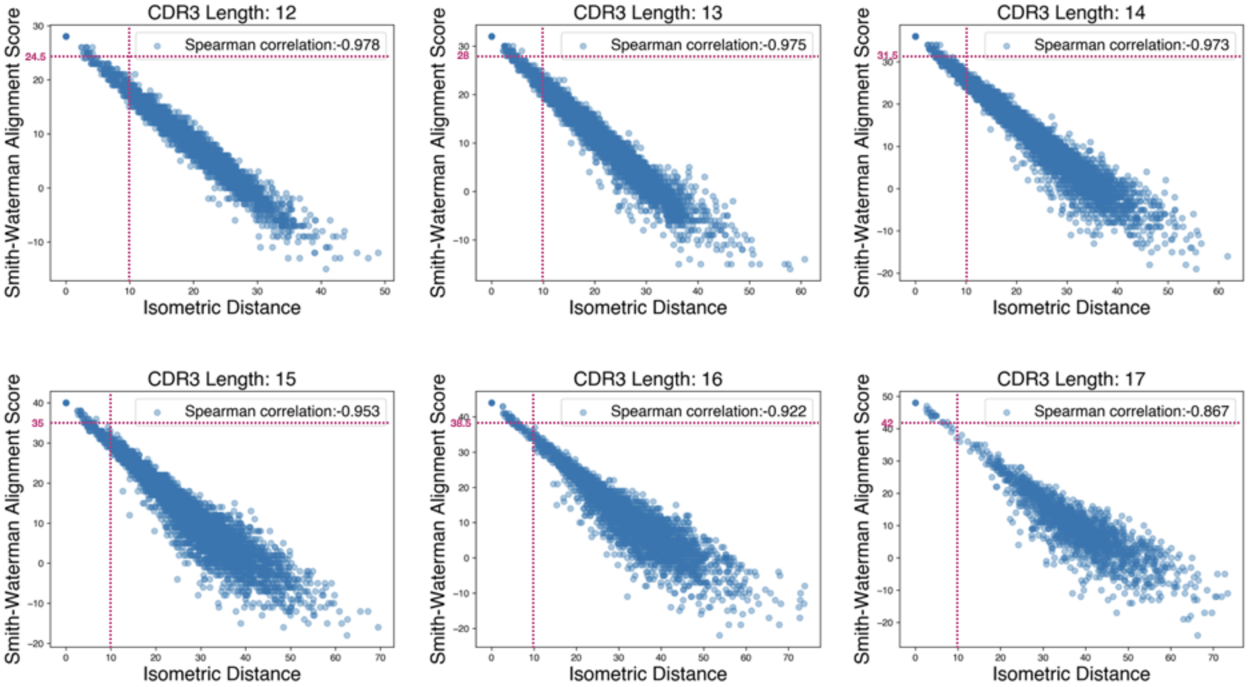

Figure S2. **Comparison of G_6-encoded isometric distances for CDR3 strings with Smith-Waterman alignment scores.** Analysis was performed for CDR3s with lengths 12 to 17. Euclidean distances (squared) between pairs of CDR3s were calculated, and compared to the corresponding Smith-Waterman alignment scores using BLOSUM62 as substitution matrix. The Spearman's correlation values were negative because higher alignment scores implicate higher similarity, which corresponded to smaller distances. This is different from the dissimilarity scores used in Figure S1. With -S option 3.5 or above, a raw Smith Waterman alignment score of $3.5 \times (L-5)$ is required to pass the clustering threshold, where L is sequence length. In each panel, horizontal lines label the position of $3.5 \times (L-5)$, where vertical lines indicating an isometric distance of 10, which is the default cutoff in GIANA.

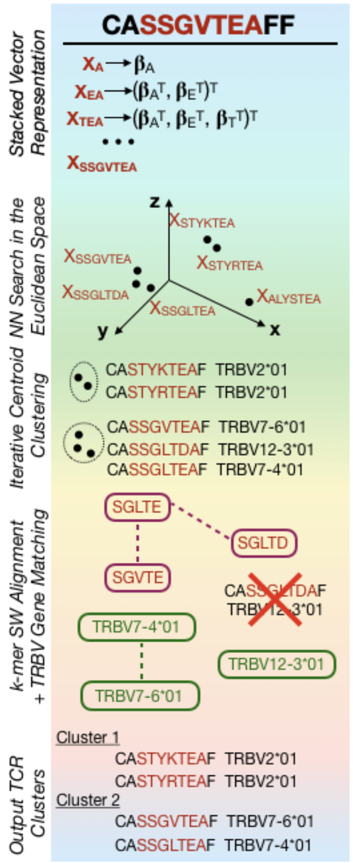

Figure S3. **Schematic illustration of stacked vector representation for GIANAsv.** For each input sequence, we concatenated the isometric vectors of each amino acid orderly to obtain a 16-by-L dimensional encoding vector, where L is the length of the CDR3 sequence. This encoding is the simplest way to preserve the isometric distance for BLOSUM62 substitution matrix. Similar to GIANA, After obtaining the encoding vectors for all the CDR3s of the same length, faiss nearest neighbor search was performed to divide the TCRs into preclusters, which were subsequently grouped into the final clusters with motif-guided SW alignment and variable gene matching.

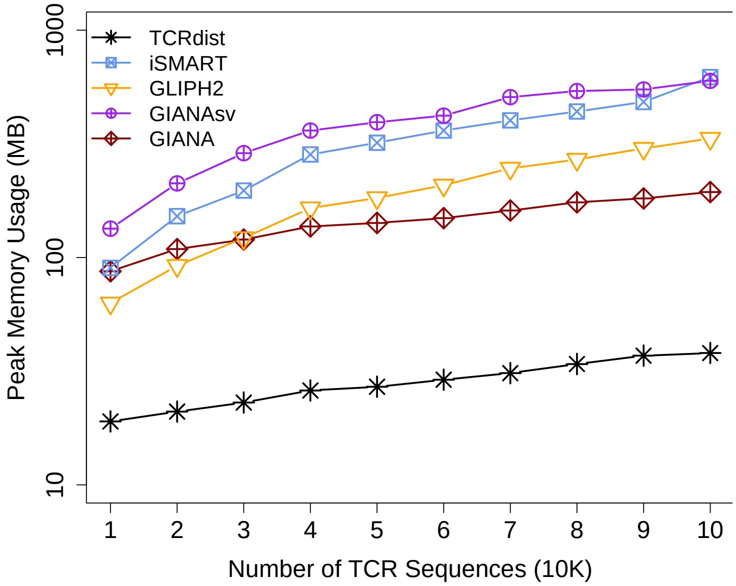

Figure S4. **Memory usage of five competing methods.** Memory allocation was estimated when evaluating time complexity in Figure 2a.

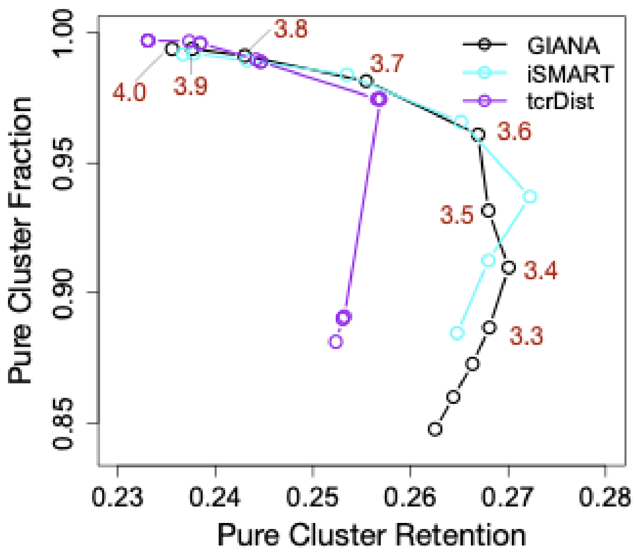

Figure S5. Parameter screening of methods using Smith-Waterman alignment in the TCR clustering. By changing the cutoff of the alignment score, for each method, Pure Cluster Fraction and Retention were calculated as described in the main text. The values for GIANA (-S option) are labeled as text labels.

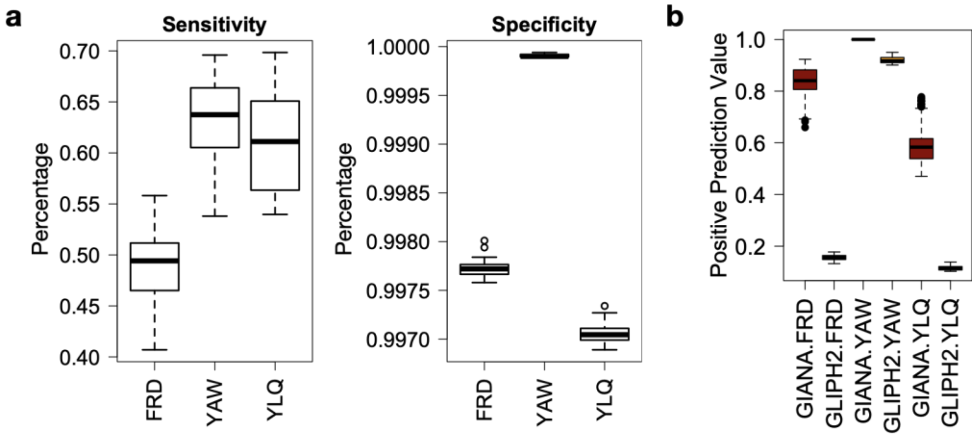

Figure S6. **Antigen-specific TCR prediction with GLIPH2**. **a**) Sensitivity and specificity estimations for GLIPH2 using the same simulated dataset as in Figure 1e. Sensitivity and specificity were defined same way as for GIANA. **b**) Positive prediction value (PPV) estimations for GLIPH2 and GIANA. PPV was defined as the total number of correctly predicted unique TCRs divided by the total number of unique TCRs clustered with the training data. "Unique" is necessary for this analysis because GLIPH2 may place one TCR into multiple clusters. For each antigen, 20 times of random sampling was performed to estimate statistical uncertainty, as shown by the boxplots.

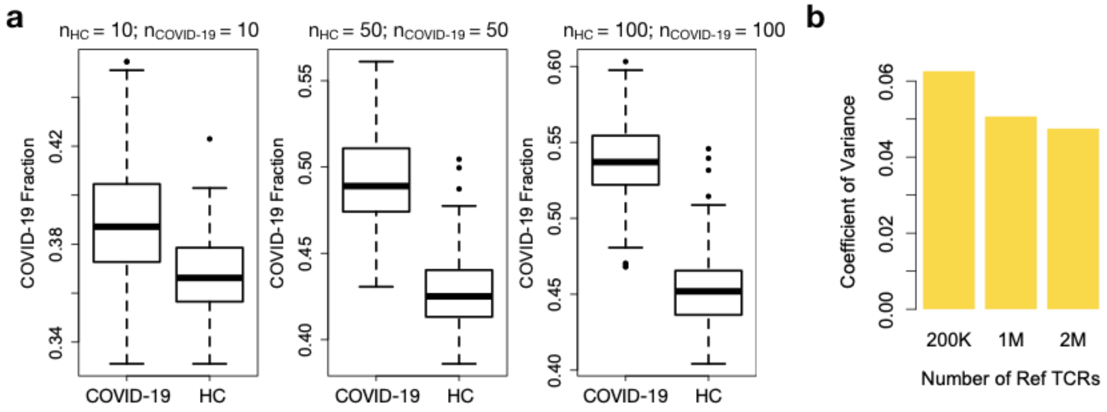

Figure S7. Coefficient of variance of COVID-19 fractions with different number of reference TCRs. **a**) Distribution of TCR fractions co-clustered with COVDI-19 reference samples under different reference data configurations. **b**) Coefficient of variance is defined as the standard deviation divided by the mean of COVID-19 fractions of the COVID-19 patients in the query samples.

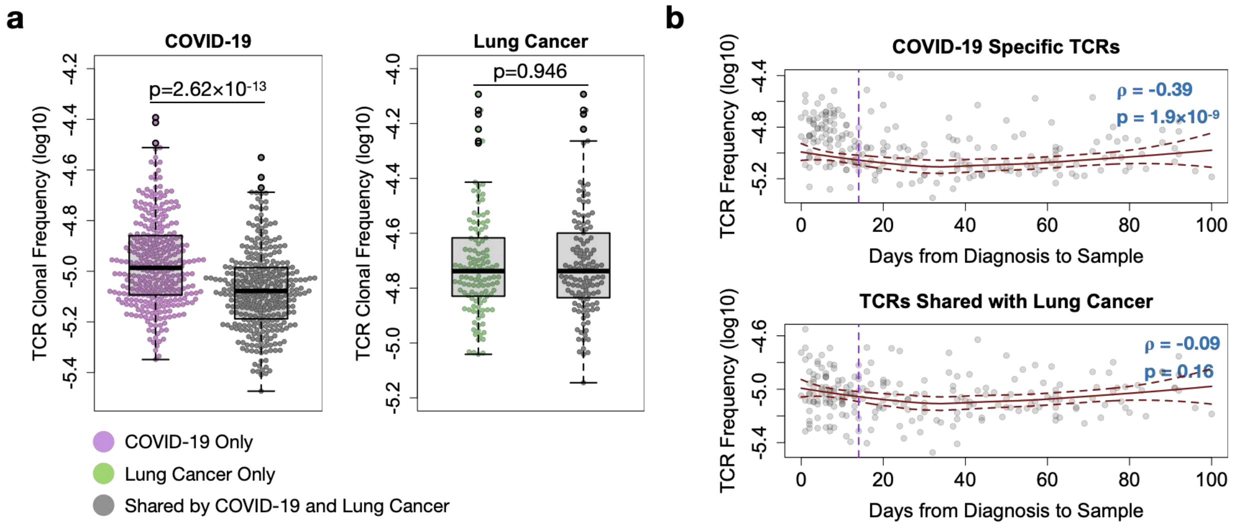

Figure S8. COVID-19 specific TCRs are dynamically regulated during virus infection. a) Beeswarm plot showing the distributions of TCR clonal frequencies of different categories. Left panel: TCRs specific to COVID-19 and those also shared with lung cancer patients. Right panel: TCRs specific to lung cancer (n=121) or shared with COVID-19 patients (n=311). For the shared TCRs, clonal frequencies were always chosen to match the cohort of the disease-specific TCRs. Two-sided Wilcoxon rank sum test was performed to estimate the p values. **b**) Dynamic changes of TCR clonal frequencies during the course of SARS-CoV-2 infection. Purple dashed line marks 14 days after the initial diagnosis. Spearman's correlation test was performed to evaluate the statistical significance between clonal frequency and time. Loess smooth curves with 95% confidence intervals were presented to visualize the trend of frequency changes.

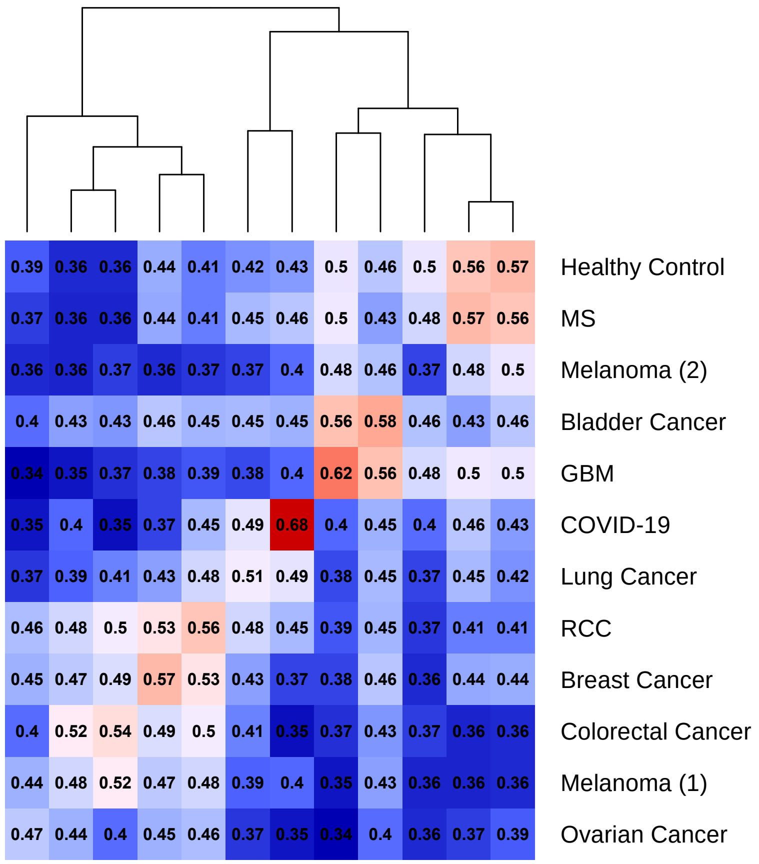

Figure S9. Cross-cohort similarity of reference TCR-seq samples. From TCR clustering data with N samples, we calculated the percentage of TCRs of each sample co-clustered with each of the other samples. We assigned the self-co-clustering percentage to be zero, to make all the vectors length N. The Spearman correlation matrix was calculated from the N-by-N co-clustering fraction matrix. The matrix is then collapsed according to the cancer types, with the mean of the top 5 highest correlations was displayed in the heatmap. Same disease correlations (diagonal values) were calculated the same way, except that self-correlations of each sample were excluded prior to the calculations.

Supplementary Tables

	TCR Number	10K	20K	30K	40K	50K	60K	70K	80K	90K	100K
GIANA	Time/s	**1.5**	**2.9**	**4.2**	**6.3**	**8.4**	**10.9**	**13.7**	**16.6**	**20.2**	**23.9**
	Memory/MB	87	109	120	137	142	149	161	175	182	194
GIANAsv	Time/s	**2.3**	**5**	**8.3**	**12.2**	**17**	**23**	**29**	**35.4**	**44**	**53.3**
	Memory/MB	134	212	288	362	394	421	508	540	549	599
iSMART	Time/s	**16.5**	**88.8**	**212**	**409**	**657**	**984**	**1380**	**1833**	**2323**	**2850**
	Memory/MB	90	152	197	284	320	362	401	439	484	622
TCRdist	Time/s	**145.9**	**580**	**1300**	**2330**	**3668**	**5411**	**7371**	**9093**	**11695**	**14338**
	Memory/MB	19	21	23	26	27	29	31	34	37	38
GLIPH2	Time/s	**16.7**	**34.9**	**51.9**	**75.1**	**99.6**	**127.3**	**156.7**	**183**	**224.2**	**271.4**
	Memory/MB	63	92	122	165	183	208	247	270	302	334

Table S1. Comparison of computational time and memory consumption of GIANA, GIANAsv, iSMART, TCRdist and GLIPH2. System configuration: macOS Catalina v10.15.2, 3.5GHz Dual-Core Intel Core i7, 16GB 2133 MHz LPDDR3.

	GIANA	iSMART	TCRdist	GLIPH2
# Clustered TCRs	17,250	16,828	18,383	31,563
# Clusters	7,586	7,649	7,316	11,945
# Pure clusters	7,289	7,387	7,130	4,333
# Pure TCRs	16,202	16,096	15,595	11.514
Specificity (Pure clusters/Clusters)	96.1%	96.6%	97.4%	36.3%
Sensitivity (Pure TCRs/Total number of TCRs)	26.7%	26.5%	25.6%	19.0%

Table S2. Evaluation of pure cluster sensitivity and clustering precision for GIANA, iSMART, TCRdist and GLIPH2. A total of 61,366 TCRs with known antigen specificity were used in this analysis. After excluding singleton TCRs (only one sequence per epitope), there were 60,700 left.

		Query TCR Number				
		10K	20K	30K	40K	50K
Reference TCR Number	200K	13	22	33	44	60
	1M	21	43	72	107	151
	2M	35	71	121	184	249
	6M	93	200	387	578	719
	10M	176	379	732	1,066	1,438

Table S3: Computational time consumption of GIANA query of TCR samples with different sizes. Time was measured in seconds.

Disease Type	Cohort	Disease	Sample Size	Unique Samples	Link	PMID
Healthy Control	Emerson et al., 2017	Healthy Control (batch1)	100	100	https://clients.adaptivebiotech.com/pub/emerson-2017-natgen	28369038
Multiple Sclerosis	Emerson et al., 2013	Multiple Sclerosis	50	25	https://clients.adaptivebiotech.com/pub/emerson-2013-jim	23428915
COVID-19	Nolan et al., 2020	COVID-19 (Adaptive, ISB)	311	311	https://clients.adaptivebiotech.com/pub/covid-2020	32793896
Cancer	Snyder et al., 2017	Bladder Cancer	117	30	https://clients.adaptivebiotech.com/pub/snyder-2017-plosmedicine	28552987
	Mansfield et al., 2018	Lung Cancer and Brain Metastasis	40	20	https://clients.adaptivebiotech.com/pub/mansfield-2018-scientificreports	29391594
	Sims et al., 2016	Glioma	32	15	https://www.ncbi.nlm.nih.gov/geo/query/acc.cgi?acc=GSE79338	27261081
	Page et al., 2019	Breast Cancer	63	16	https://clients.adaptivebiotech.com/pub/page-2019-ccr	31831558
	Reuben et al., 2019	Lung Cancer	121	121	https://clients.adaptivebiotech.com/pub/reuben-2019-natcomms	32001676
	Emerson et al., 2013	Ovarian Cancer	96	5	https://clients.adaptivebiotech.com/pub/emerson-2013-jpathol	24027095
	Stromnes et al., 2017	Pancreatic Cancer	16	16	https://clients.adaptivebiotech.com/pub/stromnes-2017-cancerimmunologyresearch	29066497
	Tumeh et al., 2014	Melanoma	34	23	https://clients.adaptivebiotech.com/pub/tumeh-2014-nature	25428505
	Le et al., 2017	Colorectal Cancer	35	3	https://clients.adaptivebiotech.com/pub/diaz-2017-science	28596308
	Duhen et al., 2018	Head and Neck, Ovarian and Melanoma	33	8	https://clients.adaptivebiotech.com/pub/duhen-2018-natcomms	30006565
	Chow et al., 2020	Renal Cell Carcinoma	53	26	https://clients.adaptivebiotech.com/pub/chow-2020-pnas	32900949
	Riaz et al., 2017	Melanoma	58	29	https://github.com/riazn/bms038_analysis	29033130
	Sherwood et al., 2013	Colorectal Cancer	14	14	https://clients.adaptivebiotech.com/pub/sherwood-2013-cii	23771160

Table S4. TCR-seq sample cohorts used as the reference data. For some cohorts, not all the available samples were used when creating the reference data. For each sample, we selected the top 10,000 most abundant TCRs, and if the data contained fewer than 10,000 sequences, all were used. Unique samples indicated the number of independent patients involved in the study. Sample size recorded the number of total TCR-seq samples in that cohort that were used in the reference. Emerson 2017 cohort contained 666 healthy donors in batch 1, from which we randomly selected 100 samples. The COVID-19 cohort contained over 1,400 patients, assembled from multiple international COVID-19 studies. We selected two cohorts collected by Adaptive Biotechnology (Adaptive, n=154) and Institute for System Biology (ISB, n-157) respectively. GIANA took 19.5 hours to cluster the reference data on a high performance computing cluster with 8 CPUs and 128G memory.

Disease Type	Cohort	Disease	Sample Size	Unique Samples	Link	PMID
Healthy Control	Emerson et al., 2017	Healthy Control (batch2)	120	120	https://clients.adaptivebiotech.com/pub/emerson-2017-natgen	28369038
	DeWitt et al., 2018	Active Tuberculosis	33	33	https://clients.adaptivebiotech.com/pub/seshadri-2018-journalofimmunology	29914888
Multiple Sclerosis	Bertoli et al., 2019	Multiple Sclerosis	12	6	https://clients.adaptivebiotech.com/pub/bertoli-2019-sr	31719595
COVID-19	Nolan et al., 2020	COVID-19 (HUniv120)	193	193	https://clients.adaptivebiotech.com/pub/covid-2020	32793896
Cancer	Beshnova et al., 2020	Ovarian, Pancreatic and Renal Cancer	25	25	https://zenodo.org/record/3894880#.YHsVai2ZN3k	32817363
	Robert et al., 2014	Melanoma	21	21	https://clients.adaptivebiotech.com/pub/robert-2014-CCR	24583799
	Beausang et al., 2017	Breast Cancer	16	16	https://clients.adaptivebiotech.com/pub/beausang-2017-pnas	29138313

Table S5. TCR-seq sample cohorts used as the query data. All 120 of the second batch of healthy donors from the Emerson 2017 study were used as control. To avoid overlap with the reference, for the COVID-19 patients, we used the Hospital Universitario 12 de Octubre (HUniv120, n=193) cohort from the Nolan 2020 study. The patients in this cohort were collected from Madrid, Spain. It took GIANA 20.5 hours to finish the query of all 420 samples on a MacBook Pro with 3.5GHz Dual-Core Intel Core i7 processor, and 16GB 2133 MHz LPDDR3 memory.
